# Effect of Variable Synthesis Conditions on the Formation of Ye’elimite-Aluminate-Calcium (YAC) Cement and Its Hydration in the Presence of Portland Cement (OPC) and Several Accessory Additives

**DOI:** 10.3390/ma16176052

**Published:** 2023-09-03

**Authors:** Karol Durczak, Michał Pyzalski, Tomasz Brylewski, Agnieszka Sujak

**Affiliations:** 1 Department of Biosystems Engineering, Faculty of Environmental and Mechanical Engineering, Poznań University of Life Sciences, Wojska Polskiego 50 Street, 60-627 Poznan, Poland; karol.durczak@up.poznan.pl (K.D.); agnieszka.sujak@up.poznan.pl (A.S.); 2 Faculty of Materials Science and Ceramics, AGH University of Science and Technology, Al. Mickiewicza 30, 30-059 Cracow, Poland; brylew@agh.edu.pl

**Keywords:** ye’elimite-aluminate cement, special binder, ye’elimite, special cement

## Abstract

In the presented study, ye’elimite-aluminate-calcium (YAC) cement was synthesized. Complete synthesis of crystalline phases was achieved at a temperature of 1300 °C, which is 150 °C lower than the temperature standardly used in the processes of obtaining calcium aluminate cements (CAC). The greatest amount of ye’elimite phase (Klein complex), roughly 87% by mass, was acquired utilizing a sulphur ion transporter derived from artificial dihydrate gypsum obtained in the flue gas desulphurization process (variation I). In the case of anhydrite, the amount of synthesized crystalline ye’elimite in the clinker was 67% by weight (variant II). Depending on the synthesis conditions in the clinkers, the quantity of obtained calcium aluminates (C_12_A_7_, CA, and CA_2_) ranged from 20 to 40% by weight. Studies on the hydration process of YAC cement samples showed that the main products are hydrated calcium aluminates and dodecahydrate calcium alumino-sulphate. In sinters of YAC and OPC, no crystalline ettringite was observed. Hydration analysis of Chinese cement revealed the presence of crystalline ettringite and dodecahydrate calcium alumino-sulphate, as well as hydrated calcium silicates of the CSH type accompanied with pseudo-crystalline Al(OH)_3_. The obtained clinkers from variants I and II constitute a special binder, which, due to its phase composition after hydration, can be used in the construction of reactors for biogas production in eco-energy applications.

## 1. Introduction

Out of the many building materials, cement is considered a strategic product dedicated to the construction industry and continues to play an irreplaceable role in the development of human civilisation. Modern cement is mainly based on the production of Portland clinker, which is a traditional yet irreplaceable building material inspired by ancient binding agents. Cement binders are widely used in construction, e.g., for housing and hydraulic engineering, as well as being very important in the construction of the country’s infrastructure and in strategic sectors related to defence and energy. Modernization of their production technology involves the introduction of improvements related to the increase in their productivity while maintaining the purity of the process and taking into consideration complex technologies related to waste disposal—an important issue from the point of view of environmental protection [1]. However, there are issues inherent in the ever-expanding cement industry, mainly due to the fact that, each year, the conventional production of Portland clinker results in the emission of approximately 3.5 Gt CO_2_-equivalent into the atmosphere [2]. Given that global cement consumption continues to increase to meet the demand for infrastructure projects and housing production, it is estimated that cement demand will grow to 4.5 Gt over the next five years, resulting in the emission of 3.9 Gt of CO_2_ equivalent [3,4]. At present, cement production accounts for approximately 8.2% of global anthropogenic CO_2_ emissions, 4.5% of which relate solely to the decarbonation of calcium carbonate. The assumptions of sustainable development and environmental protection are forcing engineers to look for technological alternatives that would result in the use of low-carbon technologies with a smaller carbon footprint compared to conventional Portland cement, which production is highly energy-consuming [4]. One possible solution is the production of special cements, in which the synthesis of the cement clinker would indicate a lower formation temperature while maintaining satisfactory physical and chemical parameters of the binders. Such cements certainly include calcium alumino-sulphate-based binders, which represent an alternative type of clinker with a lower environmental impact than Portland cement. This is evidenced by a reduction in CO_2_ emissions by between 25% and 35%, as well as lower energy requirements compared to Portland cements. These benefits are mainly attributed to the low calcium carbonate requirement and the lower firing temperature, which oscillates around 1250 °C. This is because C_3_S is not the main phase of these cements [4,5]. Cements formed in the CaO-Al_2_O_3_-Fe_2_O_3_-SiO_2_-SO_3_ or CaO-Al_2_O_3_-SiO_2_-SO_3_ systems are typically rich in a phase referred to as “ye’elimite” or “Klein” [6,7]. From the perspective of cement phase composition, a nomenclature has been proposed to categorize these cements into two groups: CSA (calcium-sulphate-aluminate), primarily composed of βC_2_S and C_4_A_3_Ŝ, and BYF (belite-ye’elimite-ferrite) cements, mainly consisting of βC_2_S and C_4_A_3_Ŝ. In BYF cements, βC_2_S forms the primary phase, accompanied by small amounts of the C_x_A_y_F_z_ phase. BYFs are also referred to as calcium sulphoglycine belite (CSAB) cements [8,9].

CSA cements stand out due to their significant potential for use as specialty binders in combination with Portland cement and calcium sulphate. On the other hand, BYF cements are considered alternatives to Portland cements, primarily because of the comparable microstructure of the solidified mixtures [6,10]. It is important to note that even a minor alteration in the chemical composition of oxide binders can lead to additional phases in the clinker’s composition, including calcium aluminates (CA, C_12_A_7_), gehlenite, anhydrite, or calcium oxide [10].

The C_4_A_3_Ŝ phase is responsible for the grout’s characteristic properties of high early strength and a short setting time, primarily due to the increased heat generated during its hydration, which is emitted during the first 12 h of the process [11]. Immediately after contact with water, C_4_A_3_Ŝ dissolves and reacts with the sulphate ions already in the liquid phase to form the main hydrated product, ettringite (AF_t_, C_6_AŜ_3_H_32_) (reaction 1).

With the depletion of calcium sulphate and dissolution, the formation of the AF_m_ (C_4_AŜH_12_) and AH_3_ (reaction 2) phases occurs. In the case of accelerated dissolution of C_4_A_3_Ŝ without depletion of CŜ and in the presence of CH, the product of hydration will also be ettringite, as shown in a simplified form (reaction 3) [12]. The hydration reaction of βC_2_S in the cements under discussion differs from its hydration in Portland cement, which forms calcium hydroxide and C-S-H. In CSA cements, βC_2_S coexists with amorphous aluminium-rich hydrates, favouring the formation of stratlingite (reaction 4) [12]. Therefore, the kinetics and products of hydration will depend on factors such as the mineralogical composition, the type and content of calcium sulphate added, and the ratio of water to cement [13].

1. C_4_A_3_Ŝ + 2CŜH_2_ + 34H→C_6_AŜ_3_H_32_ + 2AH_3_;
2. C_4_A_3_Ŝ + 18H→C_4_AŜH_12_ + 2AH_3_;
3. C_4_A_3_Ŝ + 8CŜ + 6CH + 34H→C_6_AŜ_3_H_32_;
4. βC_2_S + AH_3_ + 5H → C_2_ASH_8_.

All the cements considered are alumina-rich and therefore require raw materials rich in aluminium and iron hydroxides. Bauxite is the most commonly used source of Al_2_O_3_ and Fe_2_O_3_, but its availability is limited mainly due to its high economic cost. In order to achieve these objectives, waste raw materials can be used in the production of the above cements, which can be reused as a source of oxides in the synthesis of special cements. This approach is justified by the economics and environmental aspect of the waste treatment process. For example, waste raw materials such as fly ash, alumina-rich sludge or process-derived rea-gypsum can be used as a source of alumina or sulphate ions [14,15,16]. Research into the use of different raw materials as “waste” materials for such cements in synthesis processes is important, as the price as well as the success of the production of such cements will also depend on the price of the raw materials and their availability [17,18].

The present work concerns a study of the process of optimizing the synthesis temperature of special cements containing Klein complex. For this purpose, raw materials such as natural anhydrite and rea-gypsum, a “waste” raw material, were used as sulphate ion carriers. In this study, the influence of the proportion of different calcium sulphate carrier raw materials on the synthesis temperature and phase composition of the resulting sinters was demonstrated. Research was also conducted to evaluate the hydration processes of the sinters produced with a Klein complex and their mixtures with Portland cement.

In the course of the study, the phase composition of the hydrated slurries, the linear changes, and the compressive mechanical strength of the binders were determined [19,20]. As this work focuses on the preparation of the C_4_A_3_Ŝ phase by sintering and its application in various raw material mixtures, the individual raw materials used for the synthesis of this crystalline phase are described.

## 2. Materials and Methods

### 2.1. Sulphate Raw Materials

Anhydrite, an anhydrous calcium sulphate with the chemical formula CaSO_4_, is classified as a naturally occurring sulphate raw material. The theoretical chemical composition of anhydrite is 41.49% CaO and 58.81% SO_3_. Calcium sulphate is typically characterized by a low-defect structure, with only a few substitutions of calcium ions by Sr^2+^ or Ba^2+^ ions. Occasionally, impurities such as SiO_2_, Al_2_O_3_, MgO, or CO_2_ may occur due to the presence of heterogeneously formed crystal inclusions and/or contaminants [21].

Anhydrite crystallizes in the rhombohedral system, forming tabular crystals, occasionally elongated, and often exhibiting twinning during recrystallization. Anhydrite commonly occurs as grainy, prismatic, or fibrous aggregates with poorly defined crystal shapes, mostly appearing white in colour (though grey, blue, or colourless specimens can also be encountered). This mineral is brittle and exhibits cubic cleavage. Its hardness, according to the Mohs scale, is higher than that of gypsum, measuring 3.5, with a specific gravity of 2.96 g/cm^3^. Anhydrite is slightly soluble in water at a rate of 0.3 g/100 cm^3^ at a temperature of 20 °C [22].

Large deposits of gypsum and anhydrite were formed through the evaporation of water in enclosed marine basins and saline lakes during the Permian, Triassic, and Tertiary periods. Anhydrite precipitated from these waters when the concentration of other soluble salts was far from saturation level. It is also significant that anhydrite crystallized from the waters just prior to sodium chloride, resulting in interlayered deposits with rock-salt. Additionally, substantial anhydrite deposits were formed through the dehydration of deep-seated gypsum beds under increased pressure from overlying layers.

The most commonly used raw materials for manufacturing sulphate binders are gypsum and anhydrite rocks. However, in addition to natural raw materials, waste materials generated during the purification of sulphur compounds from flue gases in power plants, and combined heat and power plants, as well as waste from the chemical industry (e.g., phosphoric acid production, fertilizers), are also used (Table 1).

Synthetic gypsum obtained from flue gas desulphurization installations is known as desulphogypsum, technical gypsum, and energy gypsum, as well as REA gypsum or FGD gypsum (Table 2). While this material is a fully valuable raw material for the gypsum industry, it differs from natural gypsum in several properties, particularly grain size, moisture content in the material, activity, etc. [23].

Synthetic gypsum always occurs in a finely granulated form. Clear differences between synthetic gypsum and ground natural gypsum are revealed through microscopic observations. Synthetic gypsum is a powder, with the majority of particles falling within the range of 15 to 150 µm. Individual particles can take various forms, from elongated strip-like shapes to compact spherical forms. On the other hand, the particles of ground natural gypsum form irregular aggregates. The moisture content in synthetic gypsum ranges from 7% to 10%, while natural gypsum does not exceed 2%. The bulk density of synthetic gypsum, depending on the particle size, shape, and moisture content, ranges from 570 to 1270 kg/m^3^. It is worth noting that synthetic gypsum has inferior transport properties compared to natural gypsum and tends to suspend in dosing, storage, and transportation equipment.

In addition to naturally occurring gypsum and anhydrite, as well as gypsum from flue gas desulphurization processes, compounds of CaSO_4_ are also obtained as by-products in other chemical processes. An example of this is phosphogypsum (others include borogypsum, titanogypsum, and fluorogypsum), which is a waste material from the production of phosphoric acid in the fertilizer industry. The main component of phosphogypsum is calcium sulphate dihydrate formed in the reaction of sulphuric acid with phosphorites or apatites. Despite the fact that the content of dihydrate gypsum can reach 95%, the utilization of phosphogypsum as a substitute for natural gypsum is non-existent in Poland. The issue lies in the soluble compounds of phosphorus and fluorine, which prevent the use of phosphogypsum in the construction materials industry. Research has shown that phosphogypsum can be used after treatment by removing fluoride and phosphorus ions and neutralizing them with Ca(OH)_2_ [23].

Another crucial aspect from a technological standpoint is the thermal treatment of gypsum. The dehydration of calcium sulphate dihydrate is one of the most important stages in the production of gypsum and anhydrite binders. Understanding the properties of the phases present in the CaSO_4_·H_2_O system and the conditions for their formation is essential for determining how to obtain a binder with the desired properties and for determining the optimal operating conditions of the units where the dehydration process takes place.

During the heating process, gypsum gradually loses its crystalline water, transitioning first into hemihydrate gypsum and then into anhydrite. Further thermal processing leads to the decomposition of CaSO_4_ as below:

1. CaSO_4_·2H_2_O ↔ CaSO_4_·0.5H_2_O + 1.5H_2_O;
2. CaSO_4_·0.5H_2_O ↔ CaSO_4_ + 0.5H_2_O;
3. CaSO_4_ ↔ CaO + SO_2_ + 0.5O_2_.

The dehydration of gypsum and the transformation of calcium sulphate dihydrate into calcium sulphate hemihydrate is associated with a rearrangement of the crystal lattice from monoclinic symmetry to a rhombic lattice. Depending on the conditions during the dehydration process, either the α or β form of CaSO_4_·0.5H_2_O is obtained. 

The α form is produced through the dehydration of dihydrate gypsum at temperatures above 97 °C in water or in a saturated steam atmosphere. It can also be obtained by crystallization from acidic solutions followed by drying the crystals at a temperature of 40–50 °C. On the other hand, the β form is formed during the dehydration of gypsum when water vapor is removed from the reaction environment. Both forms of the hemihydrate have a rhombic structure but differ in their degree of crystallization [22,23].

The crystals of the α form of CaSO_4_·0.5H_2_O are relatively large and well-formed, and their shape largely depends on the type and concentration of soluble impurities that always accompany natural gypsum, which forms large, well-formed prismatic crystals. The β form of CaSO_4_·0.5H_2_O appears as a compact, chalky mass in which individual crystalline entities cannot be distinguished through optical observation. Both forms exhibit similar structures when examined using X-ray methods, although the β structure is more defective. Further dehydration of hemihydrate gypsum leads to the formation of anhydrite III, which is characterized by a slightly more ordered structure compared to CaSO_4_·0.5H_2_O, and exhibits high reactivity with water and the water vapor present in the air, converting back into hemihydrate gypsum. Depending on whether anhydrite III is formed from the α or β form of hemihydrate gypsum, two variations, α and β, are also distinguished [22,23].

Heating both variations of anhydrite III results in a rearrangement of the crystal lattice, an increase in its orderliness, and the formation of less reactive anhydrite II. At a temperature of 1180 °C, anhydrite II undergoes further transformation into anhydrite I, while simultaneously undergoing partial decomposition into CaO, SO_2_, and O_2_.

Sulphate materials are considered a fundamental resource for the production of a wide range of construction materials. Roasted gypsum, also known as calcined gypsum, is the most commonly used, while gypsum stone or anhydrite is only utilized in cement production.

On the other hand, anhydrite-based binders constitute a distinct group of binders derived from calcium sulphate, typically activated through grinding, calcination, and appropriate additives. This group includes anhydrite binders or “anhydrite cements” and gypsum screeds (estrichgypsum) [22,23].

#### 2.1.1. Carbonate Raw Materials

The use of carbonate raw materials is limited to the utilization of pure and extra pure limestones containing no less than 95% calcium carbonate, as well as so-called medium-grade raw materials with a calcium carbonate content below 95%. Additionally, the silica content in carbonate raw materials has a significant influence, as even a 5% content allows for the utilization of this raw material in the production of cement, such as “Chinese” type cement [24].

#### 2.1.2. Aluminous Raw Materials

The most commonly used alternative raw materials for the production of aluminous cements and those based on the Klein complex are bauxites, which serve as carriers of Al_2_O_3_ and contain a relatively low amount of iron and silica impurities. Lower grades of aluminium oxide (calcined at lower temperatures) can also be used. Waste aluminium oxides that have been used in various types of catalytic processes are also utilized [24].

### 2.2. Preparation of Experimental Variants

The implementation of the experimental part of this study was planned in several consecutive stages. The research began with determining the calcination loss in the used materials. For this purpose, three variants of approximately two grams each of the raw materials were weighed and placed in ceramic crucibles, then subjected to a temperature of 1100 °C in furnaces for a period of two hours. The calcination losses were determined based on two parallel tests and were as follows:

- Anhydrite: 2.17% H_2_O;
- Rea-gypsum: 20.02% H_2_O;
- Calcium carbonate: 31.2% H_2_O;
- Aluminium hydroxide: 33.56% H_2_O.

At the beginning of the research, two sets of raw materials were prepared, basic sets (variants I and II), which were used to obtain the Klein complex. Both mixtures consisted of calcium carbonate (CaCO_3_) and aluminium hydroxide Al(OH)_3_, but the sets differed in the addition of calcium sulphate (CaSO_4_). Specifically, the first case involved the use of gypsum, while the second case involved natural anhydrite, according to reaction schemes:

CaCO_3_ + rea-gypsum (CaSO_4_·2H_2_O)+ Al(OH)_3_—(variant I);
CaCO_3_ + natural anhydrite (CaSO_4_) + Al(OH)_3_—(variant II).

Knowing the specific calcination losses, two variants of raw materials with different qualitative compositions were prepared, which are presented in Table 3.

Additional experimental variants (III, IV) were prepared. Variant III consisted of variant I + 15% (wt.) of Portland cement “52.5” while variant IV contained “Chinese” cement based on the Klein complex (variant IV). 

**Figure 1:**
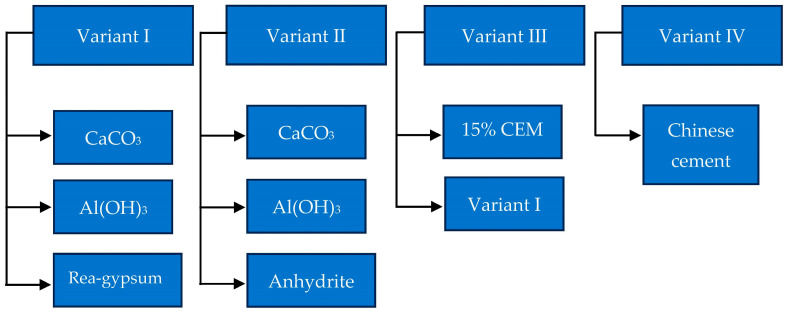
Block diagram illustrating the construction of the experimental variants.

Consequently, the collected raw materials for variants I, II, and III, forming the initial mixtures, underwent a homogenization process in a rotary mixer. The mixing was carried out for a duration of 25 h. All the experimental variants used are presented in the block diagram below (Figure 1).

### 2.3. Synthesis of the Samples

The synthesis process was tested on sets I and II after determination of the appropriate fraction (in %) of the components of each set according to stoichiometry and taking into account the obtained results of the roasting losses of individual reactants. The obtained sets were subjected to the synthesis process in platinum crucibles for half an hour using firing temperatures 1150 °C, 1200 °C, 1250 °C, 1300 °C, and 1350 °C (Czylok FCF 16/150, Jastrzębie-Zdrój, Poland). Subsequently, the samples were cooled until they reached room temperature, specifically 20 °C. The cooled samples were ground in an agate mortar following a pre-established procedure. The obtained samples were then subjected to qualitative and quantitative X-ray analysis to evaluate their phase compositions [24].

### 2.4. Hydration of Samples

The high-temperature synthesized samples that were obtained underwent a hydration process. The samples were prepared following the relevant PN-EN 196-1:2016-07 standard [25]. Prior to the phase analysis, the hydration process was interrupted using a vacuum rotary pump. The studies of the hydration process for all samples were conducted while ensuring complete repeatability of the preparatory procedures.

### 2.5. XRD Measurements

X-ray diffraction (XRD) measurements were conducted using the powder method. The samples were prepared by grinding in a vibrating agate mill (Frisch GmbH & Co. KG, Munich, Germany). Samples with a mass of 3 g were weighed (AXIS AKA320G, Gdańsk, Poland, 0.001) and ground for 1.5 h to achieve a grain size below the 0.020 mm fraction (processing time was determined experimentally). The resulting ground samples were then sieved through a mesh with a diameter of 0.020 mm (Frisch GmbH & Co. KG, Munich, Germany).

To minimize the contact with moisture, approximately 1 g of the ground samples was placed in a flat measuring holder before the XRD measurement. After completing the measurement, the measuring holder was emptied and the test portion of the preparation was placed back in a vacuum desiccator. The XRD tests were performed using a “PHILIPS” (Amsterdam, The Netherlands) apparatus consisting of a generator that supplied and stabilized the operation of the X-ray tube (PW 1140/00/60) provided with a fully modernized vertical goniometer (PW 1050/50). The goniometer was equipped with software for full automatic computer control of its operation, allowing simultaneous digital recording of measurement data. A set of equipment was assembled with a vertically mounted “PHILIPS” X-ray tube featuring a copper (Cu) anticathode and a wavelength of Kα = 1.5418 Å equipped with “Ni” filter. A “fine focus” X-ray tube PW 2216/20 with a power of 1200W and a window size of 0.4 × 8 mm was used, along with a focal spot area of 3.2 mm^2^. With an incident angle of 6 degrees, this setup allowed for the generation of a radiation beam with a width of 0.05 mm. The X-ray tube, powered by a 1000 W source, was operated at a voltage of 40 kV and a cathode filament current of 25 mA. Measurements were conducted over a fixed angular range 2θ from 10° to 65°. The Rietveld method was used for quantitative data analysis. Specialized software X’PertHighScore Plus v. 2.1 from Philips was used [26]. XRD analysis errors were calculated based on the determination of uncertainty budgets for X-ray measurements.

Quantitative analysis was performed using the Hugo Rietveld technique. The applied technique is based on the least squares method matching the profiles of the theoretical phases to the recorded diffractogram. The initial preparation of the samples was identical to that performed for qualitative analysis. In order to obtain precise diffractometric measurements that could be used for quantitative analysis, X-ray measurements were performed with the following parameters: angular range 10–90 2θ, diffractometer arm displacement step 0.02° 2θ, counting time 15 s. PANalytical-HighScore Plus v.2 software was used to analyse and interpret the obtained test results. The results of quantitative analysis were considered high quality when the GOF factor (Goodness of Fit) was less than 4. The total measurement error for quantitative analysis was ±2.5%. The used theoretical profile standards were downloaded from the Inorganic Crystal Structure Database. The detected crystal phases were characterized by the following numbers (CIF): ye’elimite—9560; CaSO_4_—40043; CaO—75786; C_12_A_7_—6287; CA_2_—14270; CA—41661 [26,27].

### 2.6. Shrinkage and Expansion Measurements

The American standard ASTM C 845–96 was used to test potential shrinkage and expansion [27]. This standard provides criteria for classifying expansive and non-shrinkage cements, along with associated standards containing methodologies for testing properties and material requirements. Some information was obtained based on the observation of changes in the behaviour of the samples and calculating results using methods available in the authors’ laboratory. Initial water requirement of the binders and the setting time were determined at the beginning, using the Vicat apparatus. Dimensional changes in the samples were measured using standard 40 × 40 × 160 mm mortar beams with empirically determined water-to-cement ratios (as per PN-EN 196-1). To compare with ASTM C-845–96 criteria, free expansion was gauged using a Graf-Kaufmann apparatus [28]. Performance property tests, conducted using standard procedures, were supplemented by assessments of phase composition and mechanical strength. Statistical analysis considered the arithmetic mean of three measurements taken on designated dates as outlined in the work schedule. The samples subjected to linear change analysis were tested 15 times (day 1 considered as starting point). Within 28 days of hydration process, 14 measurements were made (days 1 to 3, 6–7, 10, 14–17, 20, 21, 24, and 28). The last measurement was made after 90 days of the cement mortar binding process. 

### 2.7. Compressive Strength of Cement Pastes

Cement pastes for compressive strength tests were formed into cuboids with dimensions of 40 mm × 40 mm × 160 mm, in accordance with the European standard EN 196-1: 2016-07 [25]. Forms with cement pastes were left for 24 h to obtain initial strength, facilitating the maintenance of the nominal shapes of the beams. After removing the samples from the moulds, the binding process was continued for another 24 h in a climatic chamber ensuring 100% relative humidity. The samples were then placed in water saturated with calcium hydroxide.

Compressive strength tests were carried out on a compact hydraulic press (Controls S.p.A., Milan, Italy). After the samples were tested for the mechanical strength, they were stored in water saturated with calcium hydroxide and, after they were removed and dried, they were tested at specified times. The statistical analysis was performed taking into account the arithmetic mean of three measurements made for a given date provided for in the work schedule.

## 3. Results and Discussion

### 3.1. Variable Temperature Synthesis and Quantitative Analysis—Variant I

**Figure 2:**
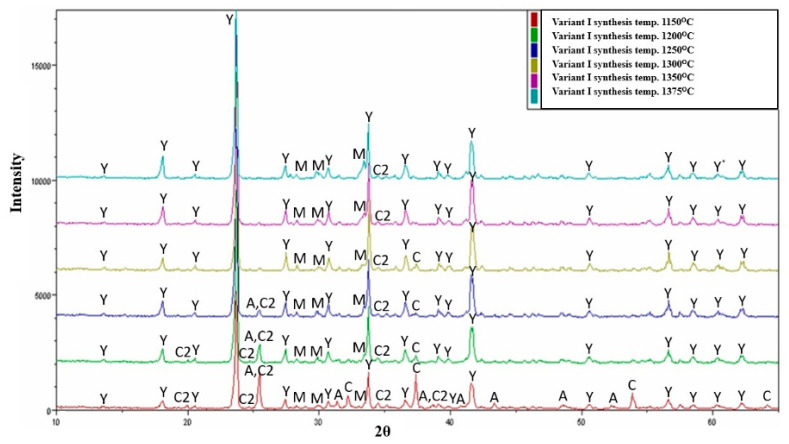
Collective roentgenograms of the variant I synthesis at different temperatures, as indicated. A—CaSO_4_·H_2_O, C—CaO, C2—CA_2_, M—C_12_A_7_, Y—C_4_A_3_Ŝ.

The analysis of the results of the sinter obtained from variant I, derived from a mixture of raw materials such as aluminium oxide, calcium carbonate, aluminium hydroxide, and rea-gypsum obtained from flue gas desulphurization, revealed the presence of diverse qualitative and quantitative composition (Figure 1 and Figure 2). The subject of the study was to obtain and synthesize the Klein phase (ye’elimite) as a component of special cement used in eco-energetic applications. However, during their realization at the specified temperatures, the analysis of the qualitative composition showed the occurrence of not only the crystalline ye’elimite (varying content) complex but also calcium aluminate phases such as C_12_A_7_, CA, and CA_2_. At the lowest applied temperature (1150 °C), the sample showed the presence of over 50% calcium alumino-sulphate phase, as well as a significant amount of unreacted anhydrous calcium sulphate, reaching approximately 14% by weight. The analysed sample also contained a noticeable amount of unreacted calcium oxide, nearly 13% by weight. Particularly interesting in the obtained research results is the presence of two calcium aluminate phases, characteristic of the phase composition of calcium aluminate cements (CAC—high alumina cements). These phases are mayenite with a content of 14% and the calcium di-glycinate with a slightly exceeding content of 6% by weight in the obtained sinter composition.

The analysis of the composition and content of individual crystalline phases in the variant I sinter indicated that, at a temperature of 1200 °C, there is a significant increase in the content of the main phase to over 75% by weight. This represents a 30% increase in its content compared to the sample obtained at a lower temperature of 1150 °C. The increase in synthesis temperature by 50 °C also results in a substantial decrease in the amount of unreacted calcium sulphate and calcium oxide, on average by 70%. At the specified temperature, there is an observable change in the quantity of calcium aluminates. The temperature increase caused a slight decomposition of dodecacalcium hepta-aluminate, which correlates with the increase in the content of the Klein complex and the decrease in the amount of free calcium sulphate and calcium oxide (Figure 2).

**Figure 3:**
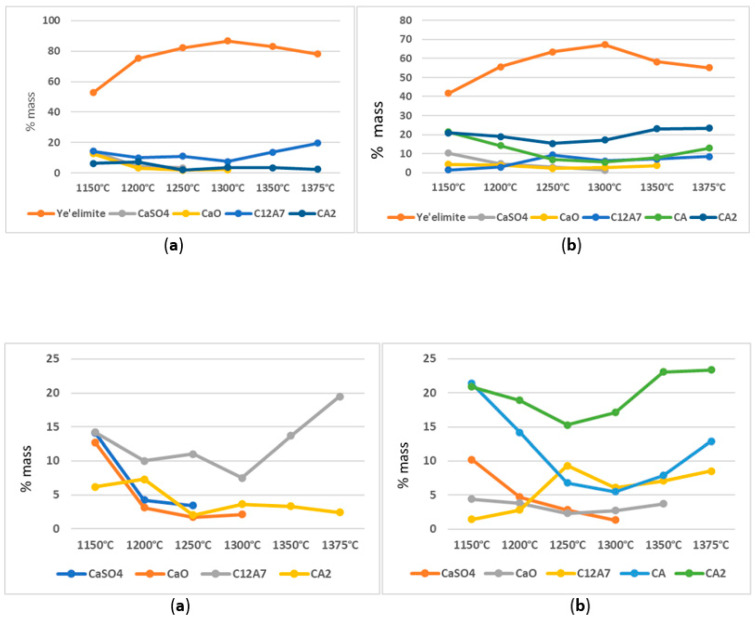
Changes in the content of crystalline phases (in wt.%) formed in variants I and II depending on the synthesis temperature. (**a**) variant I; (**b**) variant II.

In the variant I sinters obtained at a temperature of 1300 °C, the maximum content of the ye’elimite phase is achieved, with a value of 86.8% by weight (Figure 3a). The composition analysis shows no free calcium sulphate, but there are small amounts of free calcium oxide and the total content of calcium aluminates reaches 11% by weight. The temperature mentioned above seems to be optimal for obtaining the highest amounts of the crystalline ye’elimite phase. Starting from the temperature of 1300 °C, there is a slight decrease in the content of the Klein complex, while the amount of calcium aluminates increases. At temperatures of 1350 °C and 1375 °C, apart from ye’elimite, which constitutes approximately 80% by weight, and calcium aluminates, with a total value of around 20% by weight, no other free substrates are present.

### 3.2. Variable Temperature Synthesis and Quantitative Analysis—Variant II

The analysis of the results of variant II sinter made from raw materials including aluminium oxide, calcium carbonate, aluminium hydroxide, and natural anhydrite (mineral), revealed the presence of four different crystalline phases in addition to the substrates participating in the sintering process (Figure 3b). The sinter obtained at a temperature of 1150 °C is characterized by the presence of the crystalline Klein complex, which exceeds 41% by weight of the composition. Increasing the synthesis temperature of that variant to 1300 °C leads to an increase in the amount of the main phase, i.e., calcium aluminium silicate, reaching 67% by weight.

The results indicated that a variation in the synthesis temperature by 150 °C leads to an increase in the content of the Klein complex by 38% by weight, while simultaneously decreasing the content of free calcium sulphate (anhydrite) to 1% by weight. It appears reasonable to indicate that, from the perspective of the highest content of the ye’elimite phase, a temperature of 1300 °C is optimal. Further analysis revealed that the content of free calcium oxide phase in the sinter remains within the range of 4.4 to 3.7% by weight up to a temperature of 1350 °C. An important practical aspect is the presence of calcium aluminates in the phase composition, such as C_12_A_7_, CA, and CA_2_. The total content of individual calcium aluminates in the sinter fluctuates between 41% and 45% by weight with an increase in the synthesis temperature.

Anhydrite as a raw material that supplies sulphate ions in the processes of high-temperature synthesis of the YAC binder reduces the crystallization processes of higher contents of the ye’elimite phase, which can be seen in the quantitative results of the tested sample. However, it should be noted that anhydrite as a substrate in the production of special binder also leads to an increase in the content of calcium aluminates. This mechanism will be the subject of separate considerations in future works on this subject.

### 3.3. Comparison of the Degree of Synthesis and the Amount of Crystalline Phases—Variants I and II

**Figure 4:**
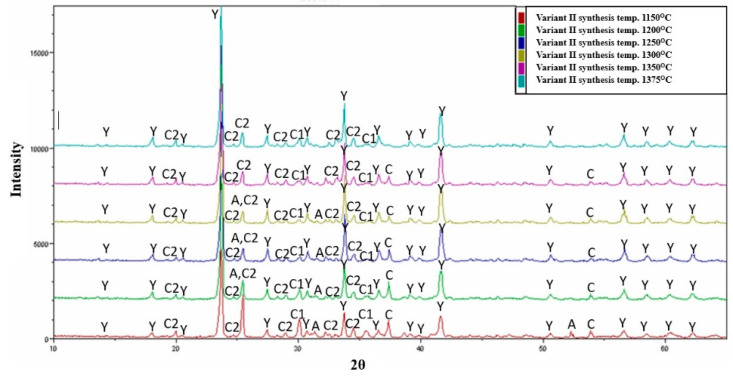
X-ray diffraction patterns for variant II at different temperatures. A—CaSO_4_, C—CaO, C1—CA, C2—CA_2_, M—C_12_A_7_, Y—C_4_A_3_Ŝ.

Comparative analysis of sinters from variants I and II revealed significant differences in the quantitative compositions of individual crystalline phases obtained from the synthesis. The analysis of diffraction patterns and curves depicting the content of the calcium aluminium silicate phase (Figure 2, Figure 3 and Figure 4) showed that their trends are similar. Up to a temperature of 1300 °C, a systematic increase in the content of the crystalline ye’elimite phase is observed and this temperature is also optimal for the synthesis of this phase. Significant differences can be observed in the amount of the obtained phase, which is dependent on the sulphate ion carrier used.

In variant I, the source of sulphate ions was rea-gypsum (dihydrate gypsum), while, in variant II, natural anhydrite (calcium sulphate without water) was present in the composition. The quantitative content of the ye’elimite phase differs between the sets by 20 to 29% by weight in favour of the sinters obtained with the involvement of rea-gypsum.

The higher amount of synthesized crystalline ye’elimite could be attributed to the fine-grained nature of the sulphate ion carrier due to the industrial conditions of obtaining rea-gypsum. Additionally, it should be noted that, during the synthesis, the sintering processes are preceded by the dehydration and dehydroxylation of gypsum, which may lead to an increase in the reactivity of sulphate ions under elevated temperatures during the formation of the Klein complex.

In both variant I and II, there is a decrease in the content (decomposition) of crystalline ye’elimite from a temperature of 1300 °C. In the case of the sinter obtained with the involvement of rea-gypsum, the decrease in content is about 10% by weight, whereas, in the sample obtained through synthesis in the presence of anhydrous calcium sulphate, the decrease in the amount of the mentioned phase reaches a value of 17% by weight. To analyse the changes in the content of crystalline ye’elimite, the curves presented in Figure 4 will be useful.

**Figure 5:**
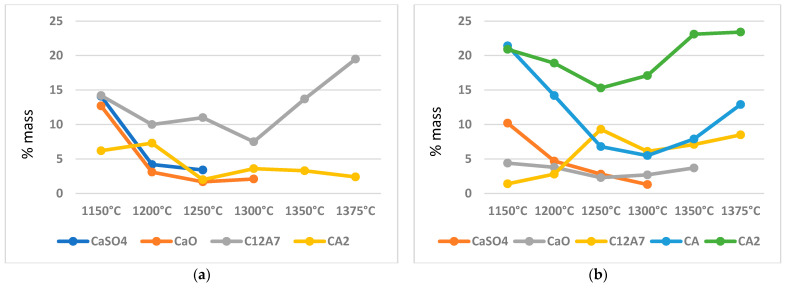
Distribution of individual (in weight %) crystalline phases, excluding the ye’elimite phase, in the sinters from variants I and II. (**a**) variant I (**b**) variant II.

The analysis of the curves in Figure 3 and Figure 5 showed a decrease in the content of the Klein complex in the sinter, which is also associated with a noticeable increase in the dodecacalcium heptaluminate phase. This is also related to a slight decrease in the amount of calcium diglycinate. In the discussed figure, the free sulphate ions that appear at lower synthesis temperatures are not visible. However, it should be noted that calcium aluminates, whose content exceeds 20% by weight in the sample, have a certain isomorphic capacity related to the ability to incorporate other ions into their structure. It is also worth noting that there is no free calcium oxide present from a temperature of 1300 °C.

**Figure 6:**
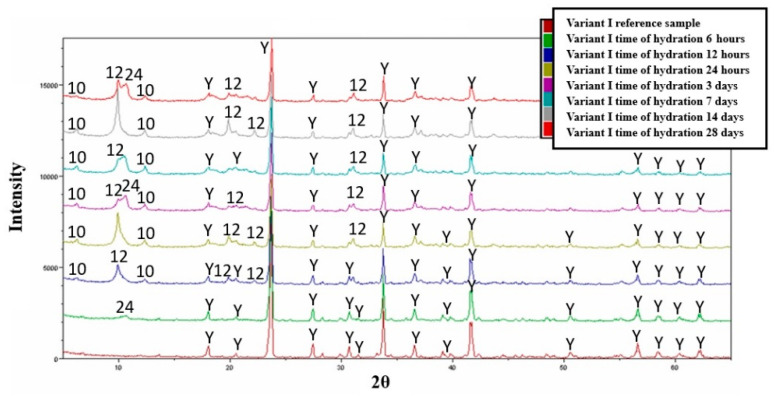
Collective roentgenograms of the variant I hydration. Y—C_4_A_3_Ŝ, 10—CaAl_2_O_4_·10H_2_O, 12—Ca_4_Al_2_SO_10_·12H_2_O or C_2_AH_6_, 24—Ca_8_Al_4_O_12_Cl_2_SO_4_·24H_2_O.

The quantitative analysis presented in Figure 6 looks different, showing a transformation in the content of individual crystalline phases. Apart from the main crystalline phase, whose trend is depicted in Figure 3, an important phenomenon is the presence of significant amounts of calcium aluminates in the sinter, exceeding the range of their occurrence in variant I.

Samples synthesized with the addition of anhydrite as a sulphate ion carrier favour the formation of significant amounts of calcium aluminates. The analysis showed that, at a temperature of 1150 °C, calcium aluminates reach almost the same percentage share, which decreases with an increase in the synthesis temperature in the direction of both crystalline phases’ lower percentage share. Starting from 1300 °C, the content of calcium aluminates present in the sinter undergoes gradual quantitative changes towards higher values. Phases CA, C_12_A_7_, and CA_2_ reach percentage shares ranging from 8.5% to 23% by weight. At the same time, on the collective curves of the ye’elimite phase in Figure 4, a decrease in its percentage share can be observed. At a temperature of 1375 °C, besides calcium aluminates and the ye’elimite phase, no other unreacted substrates are present in the sample.

In summary, the research on the thermal processing of various variants involving different calcium sulphates (rea-gypsum and natural anhydrite), calcium carbonate, and aluminium hydroxide yields interesting results, indicating that, at a temperature of 1300 °C, special cements/binders with high contents of the crystalline ye’elimite phase can be obtained. The use of waste dihydrate calcium sulphate obtained during flue gas desulphurization (rea-gypsum) leads to achieving a value of 87% by weight of the mentioned phase in the sinter, with a simultaneous high percentage of calcium aluminates, namely CA_2_ and C_12_A_7_, characteristic of calcium aluminate cements (CACs). Due to their expansive binding mechanism, these cements/binders will be suitable for special applications, including reactors used in biogas plants for biogas production [29].

The analysis of the results for the variant I samples (after 6 h) indicated that, in the initial stages of the process, the main crystallizing phases were calcium alumino-sulphates with slight doping of chloride ions—ICDD 19-203, appearing at low 2θ angles (Figure 6). This could suggest that the water used for the hydration process contained chlorine compounds that may have been incorporated into the structure of the Klein complex phase during the high-temperature synthesis in the muffle furnace due to the volatilization of its compounds [24].

### 3.4. Hydration—Variant I

The phase composition of the hydrating samples after 12 h of the process revealed characteristic products of the hydration of calcium aluminate cement, namely hydrated calcium aluminates CAH_10_ and C_2_AH_6_. The main diffraction peak of the regular hydrate (second of the hydrates) coincides with the hydrated form of dodeca-calcium alumino-sulphate. The phase composition of the tested samples does not change significantly in the later stages of the hydration process. The relative proportions of the hydration products present in the sample change, as indicated by the varying intensities of the diffraction peaks. 

In all tested samples, phases of the non-hydrated Klein complex are present. The study of the hydration process of the obtained Klein complex (variant I) provides very interesting information. According to the literature [30], the compound known as the Klein complex undergoes similar hydration to tricalcium aluminate in the presence of calcium sulphate (both are components of Portland cement), forming a highly hydrated phase in the paste, known as ettringite [31].

The Klein complex obtained through synthesis from various starting materials crystallizes during the hydration processes at different time intervals into complexly structured hydrated calcium aluminates and calcium alumino-sulphates that differ from ettringite.

### 3.5. Hydration—Variant III and Chinese Cement

Additionally, hydration studies were conducted for variant I with the addition of 15% by weight OPC (variant III) and, for comparison of the phase composition of hydrating pastes, hydration studies of Chinese cement were performed (variant IV). Chinese cements belong to the group of special cements, which include crystalline ye’elimite and calcium silicates—β C_2_S. These cements present an interesting alternative to commonly used rapid-setting binders in construction chemistry. However, the costs of their transportation are too high to widely promote their usage.

**Figure 7:**
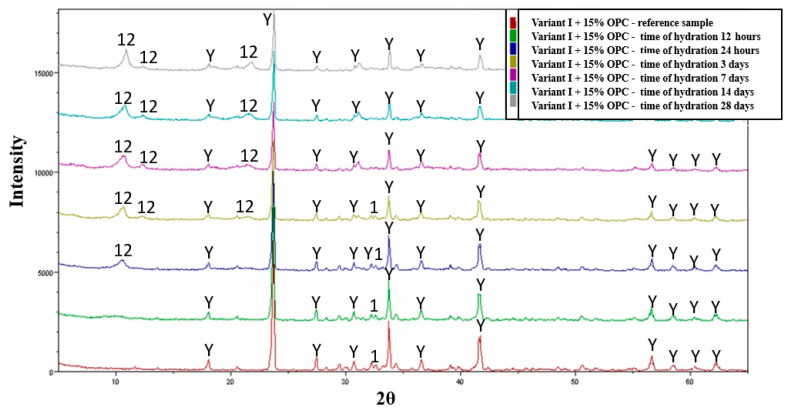
Collective roentgenograms of the variant III (variant I + 15% OPC) hydration. Y—C_4_A_3_Ŝ, 1-β C_2_S, 12—Ca_4_Al_2_SO_10_·12H_2_O or C_2_AH_6_.

The hydration studies for variant III (variant I + 15% OPC by weight) showed that the crystallization products are analogous phases to those in the variant without OPC addition, starting as early as the sixth hour of the hydration process (Figure 7). The most significant increase in the quantity of dodeca-calcium alumino-sulphate phase is observed from the 12th hour of the hydration process. In the case of the hydration of the Chinese cement, the formation of ettringite phase can be clearly observed starting from the sixth hour of the hydration process, with the most significant increase in the formation of this phase occurring between 6 and 72 h of the hydration process. With the increase in the intensity of ettringite phase reflections, starting from the 12th hour, certain amounts of dodeca-calcium alumino-sulphate begin to appear, and its quantity increases until the end of the hydration process, i.e., up to 28 days. It is also important to note that, despite the absence of reflections in the diffraction patterns, the phase composition also includes hydrated calcium silicates originating from the β C_2_S phase and certain amounts of pseudo-crystalline Al(OH)_3_ may be present.

In conclusion, it can be stated that the optimal temperature for the synthesis of special cements is 1300 °C for both raw material variants I and II. The analysis of the phase composition of the obtained sintered samples indicates that a special binder was obtained, which consists of crystalline ye’elimite and calcium aluminates characteristic of the phase composition of calcium aluminate cements—CACs.

Therefore, it can be stated that ye’elimite-aluminate cement (YAC) was obtained. It should also be noted that the temperature for the full synthesis of crystalline phases is achieved in our case at 1300 °C, which represents a reduction of 150 °C compared to the processes of obtaining CAC cements.

The hydration studies on variants I and II revealed that, due to the presence of calcium aluminates, the ettringite phase does not appear, and the paste forms phases of hydrated calcium aluminates and calcium alumino-sulphates, mainly dodeca-calcium alumino-sulphate. This relationship suggests that the presence of aluminates may lower the pH of the solution and thus hinder the formation of ettringite. The specific binding and hydration products characteristic of the obtained YAC cement become advantageous in the sense that the absence of ettringite formation will lead to an increase in the corrosion resistance of the special binder. The absence of crystalline ettringite, which contains as much as 32 water molecules, will result in a reduced water demand for the hydration process and consequently lower the overall porosity of the paste’s microstructure. This relationship indicates the potential application of the obtained special binder in the production of, for example, biogas reactors used in eco-energetics systems. So far, there have been no requirements related to the physicochemical parameters that cement should have as binders in bioreactors but the research carried out so far by the authors of the work indicates that it is possible to use YAC as a special binder in eco-energy, which will be the subject of further scientific research [32,33].

**Figure 8:**
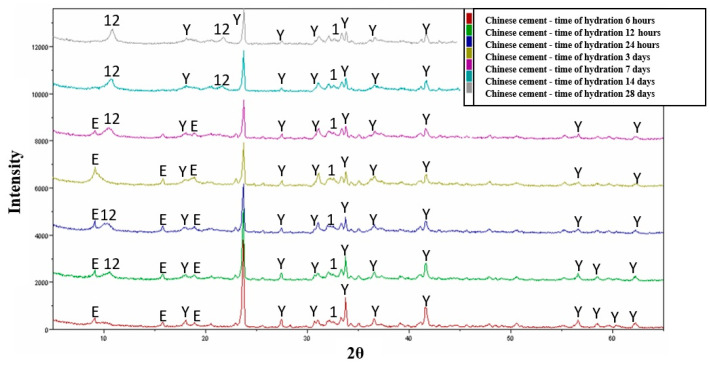
Collective roentgenograms of the Chinese cement. Y—C_4_A_3_Ŝ, E—3CaO·Al_2_O_3_·3CaSO_4_·32H_2_O, 1—βC_2_S, 12—Ca_4_Al_2_SO_10_·12H_2_O or C_2_AH_6_.

The comparison of hydration test results of YAC binder with data concerning the hydration of Chinese cement shows significant discrepancies. The hydration process of Chinese cement (Figure 8) results in the formation of a large amount of ettringite phase and, additionally, the phase composition includes hydrated calcium silicates of the CSH type derived from the β-C_2_S phase. The presence of pseudo-crystalline (gel-like) forms of Al(OH)_3_ has also been observed. From the obtained research results, it can be inferred that the key factor may be the presence of the Klein complex, which acts as an activator, providing an adequately alkaline reaction environment. This could lead to the formation of crystalline ettringite. However, it should be noted that, in all cements referred to as expansive, where the Klein complex is used, calcium silicates are usually present in their composition. Therefore, the addition of OPC (Ordinary Portland Cement) to YAC blends does not induce the formation of ettringite phases. Calcium sulphoaluminate cements (CSA or YCA) in which the main clinker composition is ye’elimite and other crystalline components are produced with significantly lower CO_2_ emissions compared to the production of Portland cements (OPC). Addition of bauxite as a raw material causes high production costs. The introduction of the addition of ye’elimite cements to clinkers could be a good way to improve their properties and reduce the ratio of clinker to cement, which will significantly reduce CO_2_ emissions [34].

### 3.6. Shrinkage and Expansion Test Results

**Figure 9:**
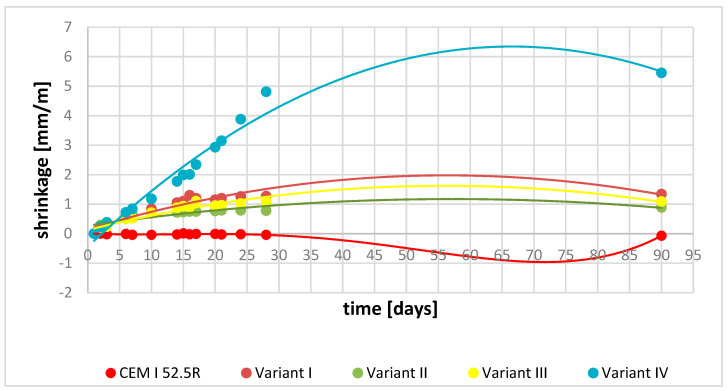
Dependence of linear changes in the sample as a function of time.

Linear changes were tested for cement mortars made of special binders obtained at 1350 °C, designated as variant I and variant II. In order to compare the results obtained on the above samples, additional tests were performed on variant III (variant I + 15% CEM I 52.5R) and variant IV—Chinese cement—(Table 4 and Figure 9). 

Analysis of the experiments on cement mortars showed the largest increase in expansion for variant IV—Chinese cement. The obtained result correlates with the phase composition determined for this variant where significant amounts of crystalline ettringite were present, which is a product of hydration of the ye’elimite phase. Such results are simultaneously confirmed by analysis of compressive strength results where a systematic increase in compressive strength was observed. The above classifies Chinese cement as an expansive binder [31,35].

The analysis of linear changes of YCA cements (variants I and II) obtained in this experiment are different compared to the studies discussed above. The obtained values are 80% lower than the value of expansion determined for Chinese cement. Going into the details of the results of the tests of YAC binders, the values of the obtained linear changes show that the mortars prepared from them have the properties of binders without shrinkage or slight expansion, which is justified by the obtained results of phase composition tests and compressive strengths. In the phase composition of hydrated YAC cements, there is no ettringite phase, which is mainly responsible for expansion. The authors of the paper justify the absence of the Candlot salt phase by the presence of significant amounts of calcium aluminates in the phase composition of special cements, which, on the one hand, improve the early strength of the slurries; on the other hand, the product of calcium aluminate hydration is also aluminium hydroxide, which, concentrating on the crystallites of the ye’elimite phase, partially blocks its further hydration [36].

However, the occurring linear changes indicate that expansive hydration products must be formed, which were not shown in the XRD analysis, most likely because they have a pseudo-crystalline structure. Significant differences between the tested samples were revealed for Portland cement marked as CEM I 52.5R. The obtained results clearly indicate the occurrence of shrinkage of the mortar made with its participation. On the other hand, the sample marked as variant III, which is a mixture of variant I and 15% CEM I 52.5R, recorded very good values of linear changes, showing the properties of a binder without shrinkage, and also having excellent mechanical strength parameters. The obtained tests clearly show that both the mixture of Portland cement with YAC and YAC binders separately have lack of shrinkage or slight expansion properties.

### 3.7. Compressive Strength Test Results

**Figure 10:**
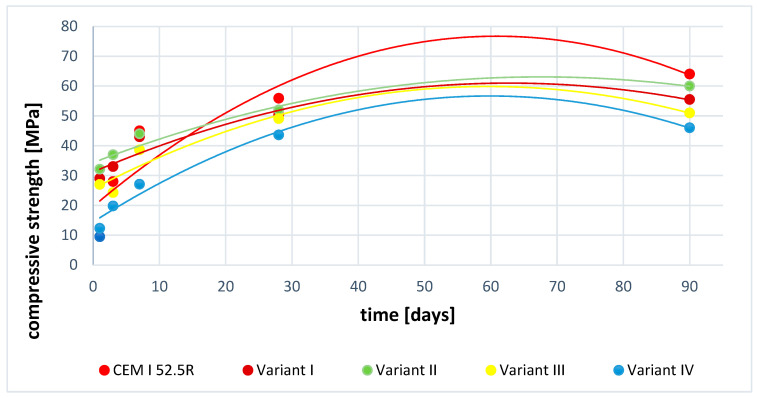
Dependence of the compressive strength of samples as a function of time.

The results of compressive strength tests were carried out for variants I and II of YAC cements obtained at the temperature of 1350 °C (Table 5 and Figure 10). The strength analysis was also carried out for a sample of Portland cement marked with the symbol CEM I 52.5R and variant III (a mixture of variant I with 15% addition of Portland cement) and variant IV (Chinese cement). The obtained results of mechanical compressive strength tests showed that the highest final values were obtained by Portland cement and its value was 64 MPa. The special binder marked as variant II obtained a 60 MPa buckle. The difference between the two binders is clearly visible in the initial stages of the cement hydration process. In the case of Portland cement, the value after 1 day was 9.5 MPa and in variant II it is 32.1 MPa. This means that the early strength of the special binder (variant II) is 70% higher than the strength recorded for Portland cement. This dependence can be explained by the presence of significant amounts of calcium aluminates in the phase composition; the mechanism of their hydration is dynamic and characteristic of high initial strengths such as those in alumina cements [21,37].

The improvement in mechanical strength is most likely related to the formation of hydrated calcium aluminate sulphates, which improve strength by densifying the microstructure due to the formation of prismatic and acicular crystallites in the cement slurry matrix. Further analysis of the obtained results shows a slight difference in the strength values between variant II and variant I, which is about 8.5% in favour of cement obtained from anhydrite.

The analysis was also performed for the mixture of variant I with the addition of 15% Portland cement. The obtained results are very interesting because they retain (with a slight systematic decrease in strength) the trend characteristic of the parameters of mechanical strength of special cements obtained in this experiment.

Interesting strength results were obtained by Chinese cement mortars (variant IV), the results of which showed a steady, systematic increase in mechanical compressive strength. The obtained results of early and final strengths recorded the lowest values in relation to other variants presented in the research work. The obtained test results showed that each of the synthesized variants is an interesting alternative to Portland cements as a binder for special applications (e.g., in conditions of biological corrosion).

The parameters of early strength are particularly important, as they give the opportunity to quickly carry out construction works in industrial conditions. The analysis also showed in each of the tested cases a systematic increase in mechanical strength, which proves that there are no changes in the microstructure of cement slurries, such as crystallite conversion, which may result in a decrease in mechanical strength [37].

From the technological point of view, the lower synthesis temperature is also important in relation to Portland cements; however, due to the costs of raw materials that are carriers of alumina (bauxites), the final production price of the special binder may be similar to OPC. Comparing the synthesis temperature, and physical and chemical parameters of YAC cements in relation to CAC cements, the economics of production of the binder presented in this manuscript can be justified.

## 4. Conclusions

Based on the conducted research and analysis of the obtained results, the following conclusions can be drawn:

1. Regardless of the type of sulphate ion carrier used (natural anhydrite and rea-gypsum), the optimal temperature for the synthesis of variants I and II is 1300 °C. Its application leads to the highest content of crystalline ye’elimite.
2. In addition to crystalline ye’elimite, the phase composition of the blends also includes calcium aluminates such as C_12_A_7_, CA, and CA_2_.
3. The main hydration products of variants I and II are hydrated calcium aluminates and dodeca-calcium-glycosulphate.
4. The hydration products of variant III, which combines variant I with 15% OPC (Ordinary Portland Cement), include dodeca-calcium-glycosulphate, hydrated silicates, and hydrated calcium aluminates.
5. In the case of Chinese cement hydration, the primary hydration product is crystalline ettringite, followed by dodeca-calcium-glycosulphate. Additionally, the phase composition includes hydrated calcium silicates of the CSH type derived from the β-C_2_S phase and pseudo-crystalline Al(OH)_3_.
6. The obtained YAC cements and their mixtures showed no shrinkage or slight expansion properties with stable results of compressive strength tests.
7. The sinters obtained from variants I and II constitute a special ye’elimite-calcium-aluminate (YAC) binder, which, due to its phase composition after hydration, is predisposed for use in the construction of reactors for biogas production in eco-energetics applications.

## Figures and Tables

**Table 1:** Chemical analysis of anhydrite from “Nowy Ląd” used for synthesis.

Component	Fraction [%]	Fraction in Terms of Calcinated Substance
% H_2_O hygroscopic at 50 °C	0.11	-
% H_2_O crystallic at 350 °C	0.94	-
% of roasting losses at 900 °C	217	-
% CaO	40.45	40.87
% MgO	0.00	0.00
% SO_3_	57.79	58.40
% Al_2_O_3_	0.07	0.08
% Fe_2_O_3_	0.00	0.00
% SiO_2_ + insoluble parts	0.64	0.65
% CaSO_4_	94.69	-
% CaSO_4_·H_2_O	4.49	-
% MgSO_4_	0.00	-
% CaCO_3_	0.00	-
**Total**	**99.89**	**100.00**

**Table 2:** Chemical composition of flue gas desulphurization gypsum from Knauf used for synthesis.

Component	Fraction [%]	Fraction in Terms of Calcinated Substance
Humidity	6.0	-
Crystallization water	20.02	-
SO_3_	45.70	57.14
CaO	32.90	41.15
MgO	0.12	0.16
Na_2_O	0.09	0.12
K_2_O	0.03	0.05
SiO_2_	0.78	1.0
Cl^-^	0.01	0.02
Al_2_O_3_	0.09	0.12
Fe_2_O_3_	0.26	0.33
**Total**	**100.18**	**100**

**Table 3:** Qualitative and quantitative composition analysis of variants I and II.

No.	Variant I	No.	Variant II
1.	CaCO_3_ *	1.	CaCO_3_
2.	Rea-gypsum (CaSO_4_·2H_2_O)	2.	Natural anhydrite (CaSO_4_)
3.	Al(OH)_3_	3.	Al(OH)_3_

* Components 1 and 3 are the same in both variants.

**Table 4:** Shrinkage and expansion analysis of variants I, II, III, IV, and CEM I 52.5R.

Sample	Flow [mm]	W/C	Linear Changes [mm/m] [days]														
			1	2	3	6	7	10	14	15	16	17	20	21	24	28	90
52.5R	172	0.45	0.00	−0.01	−0.02	−0.01	−0.04	−0.04	−0.03	−0.01	−0.02	−0.01	−0.01	−0.03	−0.02	−0.04	−0.07
V I	160	0.50	0.00	0.19	0.29	0.59	0.62	0.84	1.05	1.10	1.30	1.20	1.15	1.20	1.26	1.27	1.34
V II	165	0.45	0.00	0.29	0.28	0.64	0.66	0.69	0.72	0.73	0.75	0.75	0.77	0.79	0.79	0.78	0.89
V III	160	0.50	0.00	0.16	0.24	0.50	0.52	0.71	0.88	0.93	0.90	1.10	0.95	0.96	1.02	1.09	1.09
V IV	160	0.50	0.00	0.24	0.38	0.72	0.84	1.18	1.77	1.99	2.01	2.34	2.93	3.15	3.88	4.81	5.45

**Table 5:** Compressive strength analysis of variants I, II, III, IV, and CEM I 52.5R.

Sample	Compressive Strength [MPa] [days]				
	1	3	7	28	90
CEM I 52.5R	9.5	28.1	45.0	55.9	64.3
Variant I	29	33.0	43	50.3	55.5
Variant II	32.1	37	44	52	60.2
Variant III	27	24.4	38.6	49.1	51.1
Variant IV	12.3	19.8	27.1	43.6	46

## Data Availability

All measurement data are included in this publication. For enquiries, please contact michal.pyzalski@agh.edu.pl.
